# Nutraceuticals Inspiring the Current Therapy for Lifestyle Diseases

**DOI:** 10.1155/2019/6908716

**Published:** 2019-01-14

**Authors:** Silpi Chanda, Raj Kumar Tiwari, Arun Kumar, Kuldeep Singh

**Affiliations:** ^1^Pharmacy Institute, NIET, Greater Noida, Uttar Pradesh, India; ^2^Sanskar College of Pharmacy & Research, Ghaziabad, Uttar Pradesh, India; ^3^Department of Chemistry, MMEC, Maharishi Markandeshwar (Deemed to be University), Mullana, Haryana, India

## Abstract

Nutraceuticals are the pharmaceutically blended products that possess both nutritional as well as the medicinal value. Such a product is designed to improve the physical health, fight against day-to-day challenges such as stress, increase longevity, etc. Nowadays, emphasis is given to those herbs which are used as food and medicine due to its greater acceptance. Due to dynamic action, the popularity of nutraceuticals among people as well as healthcare providers has been increased over medicines and health supplements. This review documents herbs with a wide variety of therapeutic values such as immunity booster, antidiabetic, anticancer, antimicrobial, and gastroprotective. These herbs could be better options to formulate as nutraceuticals. Several nutraceuticals are described based on their availability as food, chemical nature, and mechanism of action.

## 1. Introduction

Hippocrates (460–377 BC), the father of modern medicine, almost 2500 years back established the relation of food and its importance for the treatment of various ailments in a very classical way optimizing various benefits [1]. Nutraceutical is composed of two words: nutrient and pharmaceutical. It is a food supplement that has a vital role in maintaining the healthy body and provides necessary supplements required for various metabolic processes to regulate body functions and thus prevents the body from diseases [1]. There is a vast cornucopia of herbs and foods which stimulate support and nourish our body system. Some have been used by different traditional systems of several countries and are now being evaluated by modern research. Use of pharmaceutical antibiotic would build up tolerances which make it ineffective in the long run. It is a better way to choose such herbs in our daily life, which would be not only capable of normalizing our body functions (even in disease condition) but also preventive and nutritive, and they also boost our immune system. An herb may not act as precisely as an antibiotic but can act as antibacterial (even antiviral) by boosting our body's own defense mechanism. To feel as a healthy well-being, one of the prominent approaches is to stay away from stress and other lifestyle diseases. The following are some examples of herbs used as food as well as medicine during infection, to boost the immune system or even in several other illnesses.

*Astragalus membranaceus* (Fabaceae) is a traditional Chinese herb. It is an extremely versatile and powerful immune enhancer antioxidant and also has hepatoprotective activity [2]. It also showed antidiabetic [3] and anticancer activity [4].

Triphala is one of the most revered tonics in Ayurveda. It is a combination of three important herbs, namely, *Terminalia bellerica* (Combretaceae), *Terminalia chebula* (Combretaceae), and *Emblica officinalis* (Phyllanthaceae). All these herbs act as a nutritive tonic. Triphala benefits almost all organs/systems of our body, particularly skin, liver, eyes, and digestive and respiratory system. The most well-known therapeutic uses are immunomodulating, antibacterial, antimutagenic, and adaptogenic, etc., which are well established [5, 6].

The northeast region of India is very rich in flora and fauna. The tribal people of the northeast region follow the principle of Hippocrates. They use their food as medicine. *Paederia foetida* (Rubiaceae) is one of the tribal plants. A research study established its gastroprotective activity and antioxidant activity [7].

The yellow powder (turmeric) from South Asia, a curry ingredient, is well known for its preventive action. It is very active against various types of bacteria, fungus, virus, and also parasite. It is a potent inhibitor of HIV [8, 9]. Asian ginseng, probably the most westernized herb, is used as a tonic. It has been popular to promote immunity [10]. The most well-known ginseng is *Panax* ginseng, and it has protective effects in neurological disorders [11].

According to Ayurveda, garlic, onion, and ginger are the basis of all healing food recipes. Garlic is one of the most widely used natural health products. These are considered as food, spice, and medicine [12].

It has been the subject of intensive study for its possible effects against heart disease and cancer [13–15]. It increases the general immune system activity. Studies have also shown to be effective in treating AIDS and antimicrobial [16–18].

## 2. Classification of Nutraceuticals

### 2.1. Nutraceuticals Based on Food Availability

#### 2.1.1. Traditional Nutraceuticals

These classes are generally sourced directly from nature, without any changes in the natural form. Various constituents such as lycopene in tomatoes, omega-3 fatty acids in salmon, or saponins in soy are available and consumed for different health benefits. Further, various types of traditional nutraceuticals are as follows:

- Chemical constituents
  - Nutrients
  - Herbals
  - Phytochemicals
- Probiotic microorganisms
- Nutraceutical enzymes

*(1) Chemical Constituents*

- Nutrients

Primary metabolites such as amino acids, various vitamins, and fatty acids had well-defined functions in various metabolic pathways. Plant and animal products along with vitamin have many health benefits and are helpful in curing diseases related to heart, kidney, lungs, etc.

Natural products obtained from plants are beneficial in treating various disorders such as brittle bones and low hemoglobin count, and they provide strength to bones and muscles, help in neuron transmission, and maintain rhythm of heart muscles. Fatty acids, omega-3 PUFAs present in salmon, had influenced the overall inflammatory response and brain function and reduced cholesterol in the arteries.

(b) Herbals

Nutraceuticals along with herbs had an excellent impact on prevention of various chronic diseases to make life better. Salicin present in the willow bark (*Salix nigra*) had been proved for anti-inflammatory, analgesic, antipyretic, astringent, and antiarthritic response clinically. Flavonoids such as psoralen present in parsley (*Petroselinum crispum*) is useful in diuretic, carminative, and antipyretic.

Peppermint (*Mentha piperita*) contains various terpenoids especially menthol, a bioactive constituent, and cures cold and flu. Tannin contents of lavender (*Lavandula angustifolia*) help releasing stress and blood pressure and are useful for lung disorders such as asthma [19].

(c) Phytochemicals

They are mainly classified on the basis of phytochemicals. Carotenoids (isoprenoids) are present in vegetables, enhancing immune system, mainly killer cells accounting for an anticancer response. Legumes (chickpeas and soybeans), grains, and palm oil contain noncarotenoids, which remove cholesterol and are anticarcinogenic.

Flavonoids, a class of secondary metabolites, which are present in most of the plants, having more than 4000 varieties had been proven clinically for preventing various diseases such as cancer, diabetes, heart diseases, and kidney problem through its antioxidant properties and their bioactive components [20].

Phenolic acids are the largest class of secondary metabolites, mainly found in citrus fruits and red wine, and have the antioxidant activity of scavenging the free radicals produced as a result of various metabolic pathways such as protein, carbohydrate, and fat. They also have anticancer and antitumour activity.

One of the classical examples is curcumin (turmeric), used as phytochemicals in most of the kitchen.

*(2) Probiotic Microorganisms*. Metchnikoff coined the term “probiotic.” Its application is well boosted in modern medicine due to its ability of making the intestine more friendly for processes such as absorption and metabolism. Probiotics are very important to make life smoother by removing the toxic flora of the intestine and maintaining a friendly environment, for example, useful consumption of *Bacillus bulgaricus* [21]. Currently various probiotic products are available in the market with adequate nutrients to counter various pathogens so that a number of ailments related to human body can be treated.

The antimicrobial property usually had an altering impact on the microflora, making the epithelial tissues more grounded and making a situation for the supplements for better retention, which is required by the body. Moreover, probiotics are very useful in lactose intolerance by the production of related enzymes (*ß*-galactosidase) and hydrolyzing lactose into its sugar components [22].

*(3) Nutraceutical Enzymes*. Enzymes are proteinous in structure, are produced by the cell, and act as a biocatalyst. It eases the metabolic rate and fastens the life process. The medical problem mainly related to the GIT whether GERD (gastroesophageal reflux disease) or constipation or diarrhoea or ulcerative colitis could be treated with enzyme supplements. The enzyme could be a better option for diabetic patients. Nowadays, enzyme therapies are used for several rare diseases such as Gaucher disease, Hunter syndrome, Fabry disease, and Pompe disease. Although enzymes are produced by their own cells, microbial sources are preferred more over plant and animal sources as they are more economical.

#### 2.1.2. Nontraditional Nutraceuticals

They are foods enriched with supplements or biotechnologically designed crops to boost the nutrients; for example, rice and broccoli are rich in *β*-carotene and vitamins, respectively. Food samples contain bioactive components which are engineered to produce products for human wellness. They are arranged as follows:

*(1) Fortified Nutraceuticals*. These types of nutraceuticals include breeding at the agriculture level or addition of compatible nutrients to the main ingredients such as minerals added to cereals, flour fortified with calcium, iron, and folic acid, and milk fortified with cholecalciferol commonly used for vitamin D deficiency [23].

*(2) Recombinant Nutraceuticals*. Biotechnology tools have been well applied through a fermentation process in various food materials such as cheese and bread to extract the enzyme useful for providing necessary nutrients at an optimum level.

### 2.2. Classification Based on Mechanism of Action

Nutraceuticals has been further classified in regard to specific therapeutic properties accounting for antimicrobial, anti-inflammatory, and antioxidant properties.

### 2.3. Classification Based on Chemical Nature

These types are classified depending upon their primary and secondary metabolite sources such as isoprenoid derivatives, phenolic substances, fatty acids, carbohydrates, and amino acid-based substances.

Different types of nutraceutical constituents of natural origin are described in Table 1. All the nutraceuticals are the resources of nature.

## 3. Conclusions

Natural products have been known for their therapeutic values for centuries. In the modern era, these substances have been used as an immunity booster; antidiabetic, anticancer, antimicrobial, and gastroprotective agents; and so on. Therefore, these herbs could be better options to be formulated as nutraceuticals.

## Figures and Tables

**Table 1:** Natural nutraceuticals along with mechanism.

Nutraceuticals	Mechanism/activity
Proanthocyanidin (chestnut fruits)	Inhibit IL-8 secretion by impairing NF-kappa-B signaling [24]
Fish-based diet	Severe osteoarthritis and hip and elbow dysplasia [25]
Curcuma extract	Decrease the level of PSA for prostate cancer [26]
Supplementation of live yeast fostered	Regulate inflammation and epithelial barrier in the rumen and express DFEB1 coding for an antimicrobial peptide [27]
Inulin-type friction dietary fiber	Immune responses against hepatitis-B [28]
Bovine milk-derived oligosaccharide and *B. lactis*	Modulate gut microbiota and immune system [29]
Lipid-based nutrient supplements	Prevent growth faltering in infants [30]
Partially hydrolyzed cow's milk proteins	Cow's milk allergy in children [31]
Lactic acid bacteria (LAB) probiotic	Endometrial inflammation and infection [32]
Lipid-based nutrient supplement (LNS)	Moderate acute malnutrition (MAM) [33]
Vitamin D supplementation	Extraskeletal benefits [34]
Neutral amino acid supplements	Optimize neurocognitive function [35]
Myo-inositol	Gestational diabetes [36]
*Lactobacillus fermentum* CRL1446	Enhances metabolism and oxidative parameters [37]
Dehydrozingerone and its dimer	Counteract the inflammation and oxidative stress [38]
25-Hydroxy vitamin D	Cognitive status in older adults [39]
Malic acid, a precursor of citrate	Antioxidant activity [40]
Combined omega-3 fatty acids	Prevents atrophy in AD-related brain [41]
*Lactobacillus rhamnosus* SP1	Insulin signaling and improves adult acne [42]
Omega-3 fatty acid ethyl esters	Breast cancer [43]
CoQ10 supplementation	Propofol inhibition on complex [44]
Omega-3 fatty acids and high-dose cholecalciferol	Type 1 diabetes [45]
Large neutral amino acid supplementation	Phenylketonuria (PKU) [46]
Low-fat yoghurt supplemented with a rooster comb extract	Muscle and joint function [47]
Lipid-based nutrient supplements	Home fortification in poor settings [48]
Cholecalciferol supplementation (HYPODD)	Arterial hypertension [49]
Omega-3 polyunsaturated fatty acid supplementation	Postmenopausal vascular disease [50]
Omega-3 fatty acids	Breast cancer prevention [51]
Myo-inositol supplementation	Gestational diabetes in obese pregnant women [52]
